# Ferroportin and Erythroid Cells: An Update

**DOI:** 10.1155/2010/404173

**Published:** 2010-08-11

**Authors:** Luciano Cianetti, Marco Gabbianelli, Nadia Maria Sposi

**Affiliations:** Department of Hematology, Oncology and Molecular Medicine, Istituto Superiore di Sanità, Viale Regina Elena 299, 00161 Rome, Italy

## Abstract

In recent years there have been major advances in our knowledge of the regulation of iron metabolism that have had implications for understanding the pathophysiology of some human disorders like beta-thalassemia and other iron overload diseases. However, little is known about the relationship among ineffective erythropoiesis, the role of iron-regulatory genes, and tissue iron distribution in beta-thalassemia. The principal aim of this paper is an update about the role of Ferroportin during human normal and pathological erythroid differentiation. Particular attention will be given to beta-thalassemia and other diseases with iron overload. Recent discoveries indicate that there is a potential for therapeutic intervention in beta-thalassemia by means of manipulating iron metabolism.

## 1. Identification, Tissue Distribution, and Structural Features of Ferroportin

**Figure 1:**
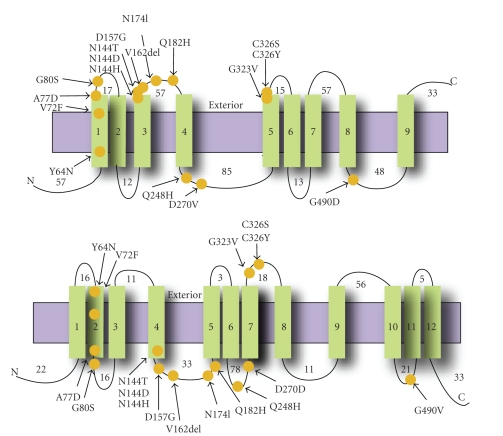
Membrane topology of FPN1. Topology of FPN1 protein is schematically represented, modified from two alternative models proposed by Devalia et al. (on the top) [16] and Liu et al. (on the bottom) [13]: the 9 or 12 predicted transmembrane helices (vertical green rectangles) are shown in relation to the lipid bilayer (horizontal violet rectangle). The positions of the mutations are marked as orange circles. The N- and C-termini are denoted by N and C, respectively. The length of extra-membranous segments is indicated.

Ferroportin (FPN1, also known as Ireg 1 or MTP1), the product of SLC40A1 gene, was independently identified by three groups, using different approaches [1–3]. FPN1 has been reported to be expressed and to play a critical role in several different tissues involved in mammalian iron homeostasis, including duodenal enterocytes (iron uptake and export into circulation), hepatocytes (storage), syncytiotrophoblasts (transfer to embryo) and reticuloendothelial macrophages (iron recycling from senescent red blood cells) [4]. FPN1 appears to act as an iron exporter [2, 3] and to be specifically regulated according to body iron requirements in these tissues [2, 4–9]. The FPN1 gene is highly conserved during evolution and encodes for a protein 571 aa in length with a predicted mass of 62 KDa [1, 3]. Sequence data showed that FPN1 is a multipass integral membrane protein iron exporter and has at least nine transmembrane alphahelices [1–3]. The locations of N- and C-termini have been largely debated in previous studies indicating for one or both termini an extracellular [10–12] or an intracellular location [13–15] (Figure 1). Different results have also been obtained for the membrane topology of FPN1 and the number of its TM domains [2, 3, 13, 16] (Figure 1). Finally, the oligomeric state of FPN1 has also been debated for several years: the protein has been reported to be a monomer [12, 15, 17] as well as a dimer/multimer [14, 18]. A recent study by using recombinant expression of FPN1 in insect cells and a biophysical characterization of purified detergent-solubilized FPN1 showed that FPN1 protein is a monomer, having 12 transmembrane regions and N- and C-termini both cytosolic [19]. In the 5′-UTR of FPN1 mRNA a putative iron responsive element (IRE) was found that could confer a translational regulation by iron regulatory proteins (IRPs) in a manner similar to other 5′-UTR-IRE-regulated genes, that is, ferritin, erythroid *δ*-aminolevulinate synthase (ALAS2) and mitochondrial aconitase [1, 20]. The 5′-UTR-FPN1-IRE was responsive to iron in HepG2 and CaCo2 cells [21]; in vitro iron deprivation inhibited translational efficiency of FPN1 mRNA [4, 6, 22]. However, the regulation of FPN1 expression by iron is currently poorly understood and a direct proof of IRP-IRE control has not been provided yet. Both transcriptional and post-transcriptional mechanisms have been implicated in the regulation of FPN1 induced by changes in cellular iron status [2, 23]. Some authors demonstrated that hepcidin, a major regulator of iron metabolism, binds to FPN1 in tissue culture cells, resulting in internalization and degradation of FPN1 and in decreased export of cellular iron [24]. The post-translational regulation of FPN1 by hepcidin may thus complete a homeostatic loop: iron regulates secretion of hepcidin, which then reduces export of cellular iron [24].

## 2. Ferroportin and Iron Overload Disorders

Ferroportin disease, or type 4 hemochromatosis, or HFE4, is an autosomal dominant condition with heterozygous mutations in the FPN1 gene [23]. Hemochromatosis associated with mutation in FPN1 can result in two different types of iron loading: one type is phenotypically indistinguishable from classical HFE hemochromatosis, in that the patients have both an elevated transferrin saturation and serum ferritin, while the other type termed “ferroportin disease” is associated with microcytic anemia, a raised serum ferritin and iron deposition in macrophages rather than hepatocytes [23]. FPN1 mutations may have three possible effects: causing misfolding of the protein and failure to reach the cell surface (“loss of function”) [10] or producing a mutant protein that is expressed at the cell surface but is not inhibited by hepcidin (“loss of regulation”) [11], or affecting iron transport ability [18]. Briefly it was shown that A77D, V162del, G490D, and D157G mutations, that are associated with typical pattern of disease in vivo, cause a loss of iron export function in vitro, but do not physically or functionally impede FPN1 protein coded by the wild-type allele [10, 11, 18] and, therefore, lead to disease by haploinsufficieny. These results are consistent with the scheme proposed by Montosi et al. [25] to explain the clinical phenotype observed in patients with these mutations [10]: lower level of serum iron, resulting from iron sequestration in macrophages, reduces availability to the bone marrow for erythropoiesis, thus leading to a middle anemia in the early stages of disease that was effectively observed in some patients and that respond poorly to phlebotomy [10, 26]. So iron overload may be a consequence of the erythron signalling to the gut enterocyte to increase iron uptake from the diet to compensate for the anemia. According to recent progress in this field it is likely that the erythron signalling is directly working through hepcidin-ferroportin interaction. By contrast the Y64N, N144D, Q248H, and C326Y mutations, which can be associated with greater transferrin saturation and more prominent iron deposition in liver parenchyma in vivo, retain iron export function in vitro [10, 11]. It was postulated that this group of mutations may resist inhibition by hepcidin, so interfering with its homeostatic negative feedback loop and resulting in a permanently “turned on” iron exporter [10, 11].

## 3. Overall View on Hematopoiesis and Importance of Iron Homeostasis during Erythroid Cells Differentiation

Iron homeostasis depends on a coordinated regulation of molecules involved in the import of this element and those exporting it out of the cells. In some cell types, such as erythroid cells, iron import mechanisms are highly expressed, thus allowing massive iron uptake [27, 28]. Excessive iron, however, may be toxic for these cells, particularly in view of its capacity to generate superoxide radicals and H_2_O_2_, which may freely diffuse into the nucleus resulting in cell damage [29] and it seemed therefore of interest to investigate whether erythroid cells possess specific mechanisms for iron export. Within the hematopoietic differentiation, the maintenance of iron homeostasis is essential for erythroid cells and macrophages. Erythroid cells need to incorporate very high amounts of iron to support the continued synthesis of heme and hemoglobin, while the macrophage cells play a key role in iron storage and recycling [30–32]. Human erythropoiesis is a dynamic complex multistep process that involves differentiation of pluripotent hematopoietic stem cells (HSCs) and early multipotent progenitors (MPPs) to generate committed erythroid precursors, the erythroblasts, which then give rise to mature erythrocytes, that is, the red blood cells (RBCs) [33–36]. Briefly, the early erythroid progenitors (BFU-E, burst-forming units-erythroid) differentiate into late colony-forming units erythroid (CFU-E) and proerythroblasts followed by a progressive wave of erythroblast maturation in polychromatic and orthochromatic erythroblasts coupled with a gradual increase of erythroid-specific markers. As the hematopoietic process progresses from the early stages into erythroid cell maturation, cells gradually lose their potential for cell proliferation and become mature enucleated cells. Mature erythrocytes are biconcave disks without mitochondria and other organelles but full of hemoglobin able to bind and deliver O_2_ [33, 37–39]. The hematopoietic differentiation is a highly complex system in which, from a pool of totipotent stem cells, originate all the cells of peripheral blood [40–43]. Developmentally, hematopoiesis in humans is characterized by three fundamental periods of activity progressively involving the yolk sac, fetal liver and bone marrow [44]. The survival, proliferation and differentiation of hematopoietic stem and progenitor cells are regulated by a complex network of hematopoietic growth factors collectively known as colony stimulating factors (CSFs), interleukins (ILs) or hemopoietins that are released from accessory cells such as fibroblasts, macrophages, lymphocytes and endothelial cells. Depending on their mechanism of action during hematopoietic differentiation, these factors can be classified into three categories: the first category includes growth factors that exert their action at the earliest stages of hematopoiesis, for example, the c-kit receptor ligand (KL) or stem cell factor (SCF) [45], FLT-3 ligand (FL) [46, 47], the basic fibroblast growth factor (bFGF) [48, 49], and interleukin-6 (IL-6) [50]; in the second category are growth factors acting as multilineage, whose prototypes are the IL-3 and GM-CSF that are able to stimulate primitive progenitors to proliferate and differentiate into all hematopoietic lineage [51]; and finally in the third category are included the growth factors acting as unilineage, that is, those that stimulate the differentiation and proliferation of a single lineage and include erythropoietin (EPO) [52, 53], the granulocytic growth factor (G-CSF) [54], monocytic growth factor (M-CSF) [55] and thrombopoietin (TPO) [56]. These unilineage factors act on progenitors already moving toward an hematopoietic lineage and promote the production of mature cells in the circulating blood, that is, erythrocytes, neutrophils, eosinophils, monocytes/macrophages and megakaryocytes. The circulating red cell mass is maintained constant by a homeostatic mechanism regulating erythropoiesis, based on an erythropoietic stimulus which ensures that, under physiological conditions, the production of red blood cells equals their destruction. Moreover, in response to hypoxia, hemorrhage or hemolysis, this stimulus causes increase in the production of red blood cells [30]. Senescent or damaged erythrocytes are phagocytized by macrophages of reticuloendothelial system that play a key role in recycling iron from hemoglobin [30].

## 4. The Presence of Ferroportin in Erythroid Cells

**Figure 2:**
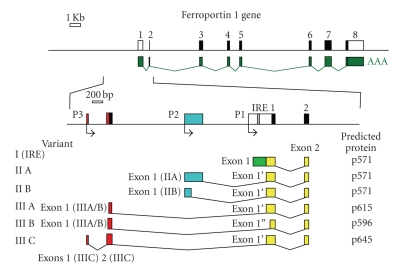
FPN1 gene structure and transcripts. Top: genomic organization and exon distribution of FPN1 (SLC40A1) locus, with (below) reported mRNA (GeneBank accession XM_047592). Middle: enlarged genomic region with exons 1-2 of FPN1 mRNA and upstream regions. Non-coding sequences are reported as open (white or coloured) boxes, coding sequences are indicated as black boxes and IRE element is indicated as a dashed box. Bent arrows below line indicate transcription start sites. P1, P2 and P3 indicate alternative promoter regions. Bottom: structure of clones obtained from 5′-RACE analysis. Alternative 5′ regions are indicated in different colours; sequences shared by all transcripts are in yellow. On the right is reported size (aa) of putative proteins coded by the respective transcripts.

**Figure 3:**
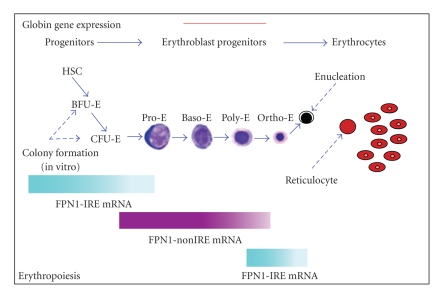
Pathway of the erythropoiesis from progenitors to mature cells. Different stages are indicated: hematopoietic stem cell (HSC), burst-forming unit erythroid (BFU-E), colony-forming unit erythroid (CFU-E), proerythroblast (ProE), basophyilic (BasoE), polychromatic (PolyE) and orthochromatic erythroblast (OrthoE). Coloured bars indicate timing of FPN1 alternative transcript expression (bottom) and hemoglobin synthesis referred to stages of erythropoiesis (top).

We reported for the first time the expression of FPN1 mRNA and protein in normal human erythroid cells at all stages of differentiation [57]. This finding was very surprising, because the presence of a transporter, that in the other tissues is appointed to export iron, was not expected for red blood cells, in which iron is believed to be all retained into the cell, committed for heme synthesis, and to exit only after death of these cells by macrophage phagocytosis. The IRE element in the 5′-UTR of FPN1 mRNA was demonstrated to be functional in erythroid cells and able to mediate translational modulation by cellular iron levels [57]. Nonetheless, FPN1 protein expression appeared to maintain a constant level during different steps of erythroid differentiation and after iron treatments [57]. This paradox would have been difficult to explain, unless of supposing the existence of a FPN1 transcript noncontaining the IRE element in erythroid cells. Actually previous studies already indicated the possibility of an IRE-independent regulation of FPN1 in different tissues and cell types, that is, iron deficiency was reported to induce in mouse, human and rat duodenum, both in vivo and in vitro, a significant increase of FPN1 mRNA expression [3, 7, 35, 58]. A nonIRE FPN1 transcript has been previously only described in polycythaemia mice as an aberrant mRNA resulting from a microdeletion in the FPN1 gene promoter [6, 59]. So, we also described for the first time the existence of two alternative FPN1 transcripts (*variant II* and *III*), other than the IRE-containing canonical one (*variant I*), that did not contain the IRE element in their 5′-UT region, did not respond to iron treatments and together accounted for more than half of total FPN1 mRNA present in erythroid cells [57] (Figure 2). These transcripts arise from the usage of alternative upstream promoters and differential splicing of 5′-UTR sequences. Interestingly, these transcripts were expressed mainly during the middle steps (4–11 days) of in vitro erythroid differentiation, corresponding to the maturation from late erythroid progenitors to polychromatophilic erythroblasts (Figure 3). At these stages of erythroid differentiation TfR1, the receptor responsible for iron incoming in erythroid cells, is strongly and increasingly expressed [28]. Therefore, the nonIRE (*variant II *and *III*) FPN1 transcripts were expressed when erythroid progenitor/precursor cells need to accumulate iron into the cells [57]. We speculated that expression of the nonIRE FPN1 transcripts could produce a constant level of the transporter, unresponsive to the very high-iron levels present in maturing erythroid cell. In contrast, IRE-containing FPN1 transcripts were mainly expressed in undifferentiated erythroid progenitors and in mature terminal erythroblasts, suggesting a possible role at these particular stages of erythroid differentiation [57]. The existence of multiple FPN1 alternative transcripts indicated a complex regulation of the FPN1 gene in erythroid cells and the possibility that the control of FPN1 expression by iron conditions in different cell types might be complex. We also speculated that in erythroid cells the regulation of FPN1 mRNA translation through the 5′-UTR IRE mechanism might be silenced because in this cell type a high level of iron uptake is needed to accumulate high amounts of iron required for optimal heme synthesis. A solution for this problem might be the utilization of an upstream alternative promoter to produce mRNA species in which the 5′-UTR IRE might be spliced out or made nonfunctional [57] (Figure 2). Our results showed that alternative FPN1 transcripts are differentially expressed during erythroid differentiation, in particular indicating a sequential and specific activation pathway, with an apparently mutual exclusion between *variant I* IRE and *variant II/III *not containing the IRE transcripts [57]. These observations suggest that erythroid precursor cells need FPN1 transcript without a IRE to evade translational control by IRP-IRE system in order to export iron during the critical period when cells are committed to proliferate and differentiate. Once the precursor erythroid cells begin to produce hemoglobin, FPN1 without a IRE diminishes and FPN1 with a IRE predominates allowing erythroid cells to limit iron export through the IRP-IRE system and synthesize heme without developing microcytic anemia. Comparison between the sequence of our variant II mRNA and aberrant nonIRE FPN1 transcript previously reported in polycythaemia mice [59] indicated a strong homology, thus strengthening our hypothesis. Recently other authors have demonstrated that also mouse duodenal epithelial cells utilized an alternative upstream promoter to express a FPN1 transcript, named FPN1B, which lacks the IRE, is not repressed in irondeficient conditions and enables duodenal enterocytes to evade translational repression [60]. Enterocyte is a particular type of cell because it must provide iron to satisfy systemic iron demands regardless of whether enterocyte itself is iron depleted [60]. The authors have so formulated a satisfactory model of why FPN1B is significantly expressed in duodenum. According to this model in iron-replete conditions both FPN1A and FPN1B transcripts are translated into FPN1 protein, which traffics to the basolateral membrane to transport iron into the circulation [60]. When the iron stores are high, the liver produces hepcidin, which causes ferroportin degradation and blocks iron absorption [24]. On the contrary in iron-deficient conditions, the liver ceases to produce hepcidin and the degradation of FPN1 is eliminated [60]. So the iron deficiency activates the IRE/IRP system which in turn blocks FPN1A translation via the IRE element. So it appears that FPN1B transcript has a key physiologic role in duodenal cells: translation of FPN1B is not repressed by IRPs allowing sufficient iron export to satisfy the systemic iron demands [60]. They also demonstrated the presence of FPN1B transcript in mouse bone marrow and in erythroid precursor MEL and G1E cell lines [60] showing a regulation comparable with our no-IRE FPN1 previously described in human, thus supporting our hypothesis that ferroportin may be subject to different regulation depending on cell type and its functions [57]. This mouse FPN1B transcript was homologous to our nonIRE variant II FPN1 transcript observed in human progenitor erythroid cells suggesting that the utilization of an upstream alternative promoter to produce mRNA species without IRE could be a physiologic and tissue-specific regulation mechanism conserved during mammalian evolution. The identification of FPN1B reveals how FPN1 expression can bypass IRP-dependent repression in intestinal iron uptake, even when cells throughout the body are iron deficient [60]. Finally, in erythroid precursor cells, they hypothesized that FPN1B expression enhances real-time sensing of systemic iron status and facilitates restriction of erythropoiesis in response to low-systemic iron in order to not create microcythemia [60]. The existence of FPN1 alternative transcripts unresponsive to regulation by IRE-IRP system in the duodenum and erythroid precursor cells suggests a cell type and tissue-specific control of FPN1 expression by systemic iron status. Previous evidence for differential effects of hepcidin in machrophages and intestinal epithelial cells can thus be explained [61].

## 5. Evidence for a Link between Erythropoiesis and Ferroportin-Hepcidin Way Regulation

In recent years there has been important advancement in our understanding of iron metabolism, mainly as a result of the discovery of hepcidin [62–64], a key regulator of whole body iron homeostasis (for an exhaustive review see [65–67]). Increasing experimental evidence suggested that a single molecule could be the “stores”, the “erythropoietic” and the “inflammation” regulator of iron absorption and recycling [68, 69], and that hepcidin acted principally or solely by binding to ferroportin, the only known cellular iron exporter, causing ferroportin to be phosphorylated, internalized, ubiquitylated, sorted [24] through the multivesicular body pathway and degraded in lysosomes [24, 70]. The aim of this review is beyond a complete picture of current knowledge on the hepcidin regulation, therefore we will focus only on those aspects that we believe influence erythropoiesis directly or indirectly. Different stimuli can modulate hepcidin and act as positive or negative regulators. At the moment we know four major regulatory pathways (erythroid, iron store, inflammatory and hypoxia-mediated regulation) that act through different signaling pathways to control the production of hepcidin. It is obvious that this complex network of interactions must be subjected to very close control in order to ensure that the iron erythropoietic demand is met and, in turn, adequate concentrations of iron in the circulation are always present (for a complete review see [66]). Under normal conditions iron store and inflammatory regulation activate hepcidin transcription in the hepatocytes through the bone morphogenetic proteins (BMPs)/SMAD4 and signal transducer and activator of transcription-3 (STAT-3) pathways, respectively [66, 71]. The hemochromatosis protein HFE, transferrin receptor 2 (TfR2) and the membrane isoform of hemojuvelin (mHJV) are all positive modulators of hepcidin transcription and when defective, lead to hemochromatosis (HH) in humans [66, 72]. Oppositely, hypoxia, anemia, increased erythropoiesis and reduced iron stores all negatively regulate hepcidin expression [66]. Emerging evidence suggests that erythropoiesis modulates hepcidin expression, with increased erythropoietic activity suppressing the action of hepcidin [73–77]. This in turn facilitates export of iron from the reticuloendothelial system and enterocytes, increasing the availability of iron for erythropoiesis [73, 76]. Anemia and hypoxia also suppress hepcidin expression, although recent experiments indicate that functional erythropoiesis is required [73, 76, 77] for these conditions to regulate hepcidin expression. We have thus reached the issue that most concerns us: erythropoiesis and iron metabolism are extremely intertwined in that alteration of one of the two may have a major impact on the second (for complete knowledge on the topic see [66, 78–83]). That is the reason why thalassemia intermedia and thalassemia major are the best studied human models of hepcidin modulation by ineffective erythropoiesis. Progressive iron overload is the most salient and ultimately fatal complication of beta-thalassemia. Iron deposition occurs in visceral organs (mainly in the heart, liver and endocrine glands), causing tissue damage and ultimately organ dysfunction and failure. Both transfusional iron overload and excess gastrointestinal absorption are contributory. Paradoxically, excess gastrointestinal iron absorption persists despite massive increases in total body iron load [68, 81, 83]. However, little is known about the relationship among ineffective erythropoiesis, the role of iron-regulatory genes, and tissue iron distribution in beta-thalassemia. If iron were a dominant regulator, patients with beta-thalassemia should express very high levels of hepcidin in serum; in contrast, the levels are very low, suggesting that the ineffective erythropoiesis alone is able to suppress the synthesis of hepcidin in spite of the presence of a severe iron overload [66, 81]. Furthermore, serum from patients with thalassemia inhibited hepcidin mRNA expression in the HepG2 cell line, which suggested the presence of a humoral factor that downregulates hepcidin [84]. The nature of the erythropoietic regulator of hepcidin is still uncharacterized, but may include one or more proteins during active erythropoiesis. Recent observations in thalassemia patients has suggested that one of these regulators could be the cytokine growth differentiation factor-15 (GDF15) [66, 85]. GDF15 is a divergent member of the transforming growth factor-beta superfamily that is secreted by erythroid precursors and other tissues. It has been identified as an oxygen-regulated transcript responding to hypoxia and as a molecule involved in hepcidin regulation [85–91]. Serum from thalassemia patients suppressed hepcidin mRNA expression in primary human hepatocytes and depletion of GDF15 reversed the hepcidin suppression [66, 85]. It was suggested that GDF15 overexpression arising from an expanded erythroid compartment contributed to iron overload in thalassemia syndromes by inhibiting hepcidin expression, possibly by antagonizing the BMP pathway [66, 85]. Without going into a detailed analysis of the GDF15 regulation mechanisms, we would like to recall the results obtained recently, that are in our view important to start reflecting on the existence of alternative ways that regulate hepcidin production. Very high levels of serum GDF15 were also observed in patients with congenital dyserythropoietic anemia type 1 (CDA I) suggesting that GDF15 contributes to the inappropriate suppression of hepcidin with subsequent secondary hemochromatosis in these patients [66, 92]. Recently a very interesting study demonstrated that expression of both GDF15 mRNA and protein was strongly and specifically responsive to intracellular iron depletion in a number of human cell lines and in vivo in humans [93]. This upregulation is independent of IRP1, IRP2 and the HIF pathway suggesting the involvement of a novel iron-regulatory pathway [93]. This study showed that GDF15 was induced by overexpression of wild-type ferroportin [93]. This observation is very intriguing because it connects the iron-mediated regulation of GDF15 concentration to patho-physiological levels of iron: despite systemic iron overload, ineffective erythropoiesis and associated iron-fluxes in beta-thalassemia might generate an iron deficiency signal in a relevant molecular or cellular context and consequent stimulation of GDF15 expression in a particular erythroid compartment [93]. Recent literatures provided at least two more molecules potentially involved in the regulation of hepcidin by erythropoiesis, that is, the human twisted gastrulation factor (TWSG1) [94] and the Oncostatin M (OsM) [95, 96]. In contrast to GDF15, the highest-level expression of TWSG1 was detected at early stages of erythroblast differentiation before hemoglobinization of the cells [94]. In human cells, TWSG1 suppressed hepcidin through a BMP-dependent mechanism [94]. In vivo studies on thalassemic mice showed that TWSG1 expression was significantly increased in the spleen, bone marrow and liver [94]. So it was proposed that TWSG1 might act with GDF15 to dysregulate iron homeostasis in beta-thalassemia [94]. In contrast to GDF15 and TWSG1, recent observations have showed that OsM could induce hepcidin expression in human hepatoma cell lines mainly through the JAK/STAT pathways [95]. Finally, results obtained by HuH7 hepatoma cells cocultered with primary human erythroblasts or erythroleukemic UT7 cells presented a 20- to 35-fold increase of hepcidin expression [96] and identified OsM responsible for increased levels of hepcidin [96]. Furthermore, this study described the biological involvement of OsM in iron metabolism “in vivo” through direct transcriptional regulation of hepcidin gene expression and suggested a new OsM-hepcidin axis that might be critical in the development of hypoferremia in inflammation [96].

## 6. Role and Regulation of Ferroportin in Erythroid Cells

The focus of this review is an update about the role of FPN1 during human normal and pathological erythroid differentiation. There is still much work to do but we think that the likely existence of alternative transcripts altered expression in all situations of ineffective erythropoiesis will give answers to unresolved issues. In erythroid cells FPN1 could be part of the signaling pathway through which the erythron communicates iron needs to expand the erythroid compartment regardless of systemic iron level. Evidence of a nonIRE FPN1 transcript in enterocytes of the duodenum supports our belief in a setting far more complex and specific to cell type. We also previously demonstrated the existence of a FPN1 variant IIIA alternative transcript with the potential to code for a longer protein with 44 additional amino acids (p615), and a FPN1 variant IIIC alternative transcript that potentially coded for a long protein with 74 additional amino acids (p645), both N-terminal to and in frame with the canonical open reading frame [57] (Figure 2). Only FPN1 variant 1-IRE transcript has an IRE sequence in the 5′UTR, whereas all the other transcript types do not. Unfortunately all these transcripts differ by 100-200 bp in length and cannot be easily detected as distinct bands in Northern analysis [57]. Interestingly, also these transcripts were expressed mainly during the middle steps 4–11 days of in vitro erythroid differentiation, corresponding to the maturation from late erythroid progenitors to polychromatophilic erythroblasts [57] (Figure 3). Therefore, the nonIRE (*variant II and III*) FPN1 transcripts were expressed when erythroid progenitor/precursor cells need to accumulate iron in the cell [57]. At the moment we do not know yet whether the hypothetical isoforms p615 and p645 are actually present in vivo because of the difficulty of obtaining specific antibodies. As mentioned earlier, to explain the surprising finding that FPN1 protein expression was not responsive to iron conditions although about 50% of FPN1 is encoded by the IRE transcript, we speculated that in erythroid cells the regulation of FPN1 mRNA expression through the 5′-UTR IRE mechanism might be silenced because in this cell type is needed to accumulate large amounts of iron for optimal heme synthesis [57] and a solution to this problem could be to use an upstream alternative promoters to produce mRNA species in which the 5′-UTR IRE could be spliced out or made nonfunctional. Several studies support our hypothesis: a recent work has shown an high frequency of alternative first exons in erythroid genes suggesting a critical role in regulating gene function [97]. In the opinion of the authors the frequent presence of consensus translation initiation sites among the alternative first exons suggests that many proteins have alternative N-terminal structures whose expression can be coupled to promoter choice [97]. So it seems that first exons and alternative promoters are more widespread in the human genome than previously appreciated and that they may play a chief role in regulating expression of selected protein isoforms in a tissue-specific manner [97]. Recently it was also demonstrated that many non productive transcriptional initiation events occurred in the vicinity of established promoters, some of which may produce mRNAs with altered translation efficiency, allowing transcripts to evolve to meet specific physiological needs [98]. In conclusion we would like to emphasize that the presence of alternative ferroportin transcripts without an IRE in erythroid cells leaves open the possibility that alterations in ferroportin mRNA splicing may be relevant in pathological conditions of altered erythroid differentiation [57, 59, 99]. So it would be interesting to investigate the possibility of regulatory mutations in various iron disorders, in particular when type 4 hemochromatosis is present in the absence of coding region mutations or in all cases of familial hyperferritinemia and in sporadic cases in the absence of known secondary causes (i.e., inflammation, malignancy infection or dysmetabolism) where “ferroportin disease” should be suspected. We previously demonstrated that FPN1 protein appeared to be localized at the level of the cytoplasm both in vesicles and in the cytosol in erythroid cells, suggesting that FPN1 may be involved in the intracellular trafficking of iron between the cytosol and organelles [57]. In contrast to the clear detection of FPN1 at the basolateral membrane of enterocytes, immunofluorescence studies with macrophages revealed a pronounced vesicular and mostly intracellular localization [100]. In particular confocal analysis revealed the presence of some FPN1 microdomains at the plasma membrane, likely suggesting a vesicular trafficking of the protein between the cytosol and cell surface [100]. FPN1 might be stored within the cell until it is needed for iron export, at which point it might be recruited to the membrane [100]. Alternatively, FPN1 might mediate iron export through the use of an intracellular vesicular compartment, in which FPN1 would act as an iron “concentrator” [100]. Such a vesicular compartment could then be recruited to the plasma membrane via exocytosis [100]. Furthermore, although proteins required for heme biosynthesis and Fe-S cluster assembly have been identified, we know little about intracellular iron trafficking, particularly to mitochondria. We do not exclude the possibility that FPN1 protein may be involved in this pathway of iron metabolism. Some authors have demonstrated that heme derived from human or murine red blood cells or from an exogenous source of heme led to marked transcriptional activation of the FPN1 and HO1 genes [101]. Furthermore, the iron released from heme catabolism subsequently stimulated the expression of ferroportin mRNA and protein, indicating the existence of a dual mechanism of ferroportin regulation in this cell model, characterized by an early induction of gene transcription mediated primarily by heme, followed by a post-transcriptional regulation iron mediated [101]. So it is therefore tempting to speculate that similar regulatory mechanisms could be involved in the transcriptional regulation of FPN1 by heme in erythroid cells. Besides its function as prosthetic group in heme proteins, heme itself can influence gene expression at the level of transcription, protein synthesis, microRNA processing or post-translational modifications. Heme is a potent inducer of heme oxygenase 1 (HO1), a cytoprotective and anti-inflammatory molecule which catalyzes heme degradation. Heme inactivates the transcriptional repressor Bach1, thereby allowing the binding of Nrf2 to Maf recognition elements (MAREs) present in the regulatory regions HO1 [102, 103]. MAREs are also present in the enhancer of the H ferritin gene [102, 104] or in the ß globin Locus Control Region [102, 105]. Although HO1 expression has not been extensively studied in erythroid cells, it has been shown that HO1 mRNA decreases following erythroid differentiation of Friend erythroleukemia cells, while mRNAs coding for the enzymes of the heme biosynthetic pathway increase [102, 106]. Furthermore, it was previously reported that heme mediated derepression of Maf recognition element through direct binding to transcription repressor Bach1 [107]; Nrf2 transcription factor regulated induction of the heme oxygenase-1 gene [108] and coordinately regulated a group of oxidative stress-inducible genes in macrophages [109]; Bach1 was a sensor of cellular heme levels [110]. A very recent study has showed that heme controlled the transcription of the iron exporter FPN1 involving Bach1 activity, Nrf2 nuclear accumulation and a highly conserved MARE/ARE enhancer element located at position −7007/−7016 of the murine FPN1 promoter in macrophages [111]. This suggest that iron recycling from heme involves a single transcription control mechanism that regulates heme catabolism, iron storage and detoxification as well as iron export in a coordinated manner [111].

## 7. New Potential Therapeutic Approaches

It is increasingly evident that the iron metabolism, heme and cellular erythropoiesis are inextricably linked, because iron metabolism [71, 112] and cellular heme (for exhaustive review see [113]) are two of the most relevant key regulators of erythropoiesis. The complex regulation of erythropoiesis suggests the existence of several molecular targets that could be exploited therapeutically for treatment of RBC disorders like thalassemias and anemias [33]. We must differentiate between primary iron overload, and iron overload that accompanies ineffective erythropoiesis: in the latter case the administration of hepcidin might be considered as a new potential therapeutic approach to reduce iron overload in thalassemias and other forms of anemia associated with ineffective erythropoiesis [33]. The reduced number or the absence of mature erythroid cells in beta-thalassemia patients is still very difficult to understand, and it has become one of the paradoxes among the most difficult to resolve (as noted Stefano Rivella in one of his very comprehensive review [83]): when the body has greater need for red blood cells instead it responds by decreasing their production. The most probable hypothesis to explain this phenomenon might rely on the existence of intrinsic and extrinsic mechanisms that would affect the process of differentiation: for example in cells where the synthesis of beta-globin gene is defective to the point that they ensure a stoichiometric between alpha and beta globin chains, a security mechanism can block the intrinsic maturation or, alternatively, an amount of heme in excess can be an extrinsic signal to prevent the differentiation that would lead to clusters of alpha globin chains production of reactive oxygen species (ROS ) too toxic to survive [83]. There is much experimental evidence that oxidative stress may limit the process of differentiation. All this of course worsens the anemic outline [83]. So the contribute of these mechanisms to ineffective erythropoiesis might be different in each patient according to level of beta-globin synthesis and other extrinsic factors such as iron overload [83]. At this point the question arises: is there a meeting point between different signaling pathways, although activated by different signals? Recent discoveries indicate that there is a potential for therapeutic intervention in beta-thalassemia by means of manipulating iron metabolism [80, 83, 114]. A recent study suggested a link between EpoR/Jak/Stat signaling and iron metabolism, showing that in mice that completely lack Stat5 activity the cell surface levels of TfR1 on erythroid cells were decreased more than 2-fold [115]. Another study suggested a direct involvement of Epo in hepcidin regulation through the transcriptional factor C/EBP alpha [116]. In addition it has been shown a link between Jak 2 and FPN1: Jak2 phosphorylates FPN1 following binding of this protein to hepcidin [117]. Phosphorylation of FPN1 then triggers its internalization and degradation [117]. Therefore Jak2 might represent one of the major links at the interface between erythropoiesis and iron metabolism suggesting that use of Jak2 inhibitors, antioxidant, and analog of the hepcidin might be used to reduce ineffective erythropoiesis and abnormal iron absorption [83]. Administration of synthetic hepcidin or of agents that increase its expression, may be beneficial in controlling absorption of this metal [66]. Also GDF15 could be another potential therapeutic target for beta-thalassemia syndromes [85]. A major goal of hemoglobinopathy research is to develop treatments that correct the underlying molecular defects responsible for sickle cell disease and beta-thalassemia [114]. One approach to achieving this goal is the pharmacologic induction of fetal hemoglobin (HbF) [114]. Although many of the events controlling the activity of the beta-globin locus are known, the details of those regulating normal human hemoglobin switching and reactivation of HbF in adult hematopoietic cells remain to be elucidated. If the molecular events in hemoglobin switching or gamma-globin gene reactivation were better understood and HbF could be fully reactivated in adult cells, the insights obtained might lead to a cure for these disorders. Agents that increase human HbF in patients may work at one or more levels: for example, hydroxyurea and 5-azacytidine kill dividing cells preferentially and may increase gamma-globin expression indirectly through this effect (for complete reviews see [114, 118]). Butyrate may work both by HDAC inhibition and by increasing gamma-globin translation on ribosomes [114, 118]. The Stem Cell Factor (SCF) induced an “in vitro” expansion of effective erythropoiesis and a reactivation of gamma-globin synthesis up to fetal levels, paving the way to its potential use in the therapeutic treatment of this disease [119]. Recently it was reported the ability of thalidomide to increase gamma-globin gene expression and the proportion of HbF-containing cells in a human in vitro erythroid differentiation system [120] showing that thalidomide induced production of ROS that in turn caused p38 MAPK phosphorylation and globally increased histone H4 acetylation [114, 120]. All these experiments present a body of evidence that suggests an important role for intracellular signaling in HbF induction [114]. Finally recent publications have demonstrated the importance of what has been termed the “integrated stress response” pathway in erythroid cells that is also activated from a variety of stress stimuli, including viral infection, NO, heat shock, ROS, endoplasmic reticulum stress, ultraviolet irradiation, proteosome inhibition, inadequate nutrients and, in erythroid cells, limiting amounts of heme [114, 121, 122]. In conclusion we are increasingly convinced of the importance to study the molecular mechanisms of iron homeostasis dysregulation in thalassemia and in particular the GDF15-BMP-Hepcidin-Ferroportin regulatory way in order to understand its contribution to iron overload.
